# Effectiveness of the Ultrasound-Guided Interfascial Injection Applied in Addition to Physical Therapy Applications in Chronic Low Back Pain: A Quasi-Experimental Study

**DOI:** 10.5152/eurasianjmed.2024.23286

**Published:** 2024-02-01

**Authors:** Burcu Metin Ökmen, Korgün Ökmen

**Affiliations:** 1 Department of Physical Medicine and Rehabilitation, Bursa Yüksek İhtisas Training and Research Hospital, Bursa, Turkey; 2 Department of Anesthesiology and Reanimation, Bursa Yüksek İhtisas Training and Research Hospital, Bursa, Turkey

**Keywords:** Chronic pain, local anesthetic, low back pain, pain, steroid, thoracolomber fascia

## Abstract

**Background:**

Anatomical and histological features of the thoracolumbar fascia may play an active role in chronic low back pain (LBP). This study aimed to evaluate the efficacy of interfascial injection in patients with LBP.

**Methods:**

Sixty participants with chronic LBP were recruited for this study. The patients were allocated to 2 groups: physical therapy (PT) (n = 30) and PT + interfascial injection (IFI) (n = 31, 10mL (0.25% bupivacaine) + methylprednisolone (40 mg) injection into the middle layer between the quadratus lumborum and erector spinae muscle). Outcome measures involved performing Numeric Rating Scale (NRS) and Oswestry Disability Index (ODI) scoring on study participants at pretreatment (PRT), as well as posttreatment at months first, second, fourth, and sixth.

**Results:**

In both groups, NRS and ODI scores were statistically significantly lower than PRT values at the first, second, third, fourth, and sixth months. (*P *< .05) NRS and ODI scores were significantly lower in the IFI and PT groups compared to the PT group at the first, second, fourth, and sixth months. (*P *< .05).

**Conclusion:**

***: *** The study result shows that IFI applied to the middle layer of the thoracolumbar fascia may be effective in individuals with chronic LBP. The effect of fascial structures on LBP should be further investigated.

**Main Points**

- The structures that may cause low back pain (LBP) are connective tissues, muscles, nerve roots, joints, vertebrae, and the thoracolumbar fascia (TLF).
- There is important data identifying the TLF as an anatomical and histological source of pain.
- Interfascial injection can be a helpful method in the treatment of LBP.

## Introduction

Low back pain (LBP) refers to pain that is not related to a specific medical condition (osteoporosis, fracture, inflammatory disorder, radicular syndrome, etc.).^1^ Chronic low back pain can arise from various factors. Among these, intervertebral disc disorders are prevalent in clinical practice.^1^ Previous studies have implicated inflammatory processes along with mechanical compression in the etiology of LBP. Inflammation in the nerve roots mediates the occurrence of pain.^2,3^ Accordingly, patients diagnosed with lumbar disc herniation can benefit from conservative therapies such as bed rest, medical therapies (NSAIDs), physical therapy (PT), and exercises. The available literature has not yet defined the exact cause of LBP or a treatment aimed at alleviating this cause.^3^ One perspective suggests that any area within the lumbar region’s anatomy can be a source of LBP. The structures that may cause LBP are connective tissues, muscles, nerve roots, joints, vertebrae, and the thoracolumbar fascia (TLF).^4-7^ The TLF, as one of the implicated tissues in LBP, is also one of the structures with the highest sensitivity to chemical stimulation, and so is considered to be important in non-specific LBP.^4^ Despite the paucity of studies, there is important data identifying the TLF as an anatomical and histological source of pain.^5-7^ Fascial plane injections involving the injection of local anesthetics amid the fascial structures have entered into common use in recent years.^8^

The hypothesis of our investigation was that the combination of interfascial injection (IFI) and PT would effectively treat chronic low back pain. In this study, we aimed to determine the effectiveness of the IFI used in addition to the PT methods with the Numeric Rating Scale (NRS) and Oswestry Disability Index (ODI) scores at 6 months.

## Material and Methods

**Figure 1.**
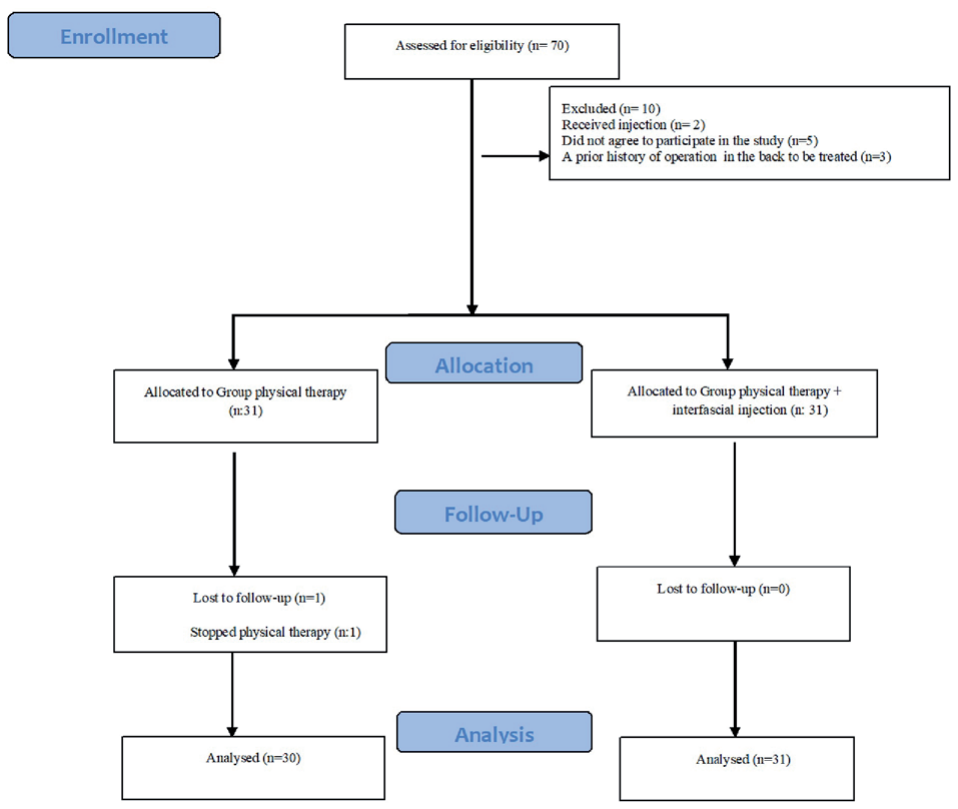
Flow Diagram.

A total of 70 participants aged between 18 and 65 years with a history of chronic LBP were recruited for this quasi-experimental, prospective study following ethical from Bursa Yüksek İhtisas Training and Research Hospital (ethics committee approval number: 2019/10-24). The present study adhered to the ethical guidelines outlined for the conduct of biomedical research using human subjects in the Declaration of Helsinki. Each patient who agreed to undergo the IFI procedures gave written informed consent. Inclusion Criteria: Patients must have experienced LBP for a minimum of 6 months, exhibit magento resonance findings diagnosing lumbar disc disease (LDD), and present with a NRS score exceeding 5. Exclusion Criteria: Patients who have previously undergone lumbar or spinal surgery and those diagnosed with spondylolisthesis or spinal stenosis were excluded from the study. The study participants who volunteered were allocated to 2 groups: Group PT (n = 30) and Group PT + IFI (n = 31) (Figure 1).

## Treatment Protocols

Physical therapy: All participants underwent the same PT treatment, which included a 20-minute session with a warm compress, 6 minutes of ultrasound (1 MHz frequency and 1.5 W/cm^2^ intensity), and specially designed therapeutic exercises aimed at the lower back muscles. These exercises included stretching, hyperextension, bridge, posterior pelvic tilt, and cat-camel movements and were taught to patients by a specialist with extensive experience. Patients were directed to conduct 2 sets of exercises daily, each set comprising 5 repetitions of all motions.

**Figure 2.**
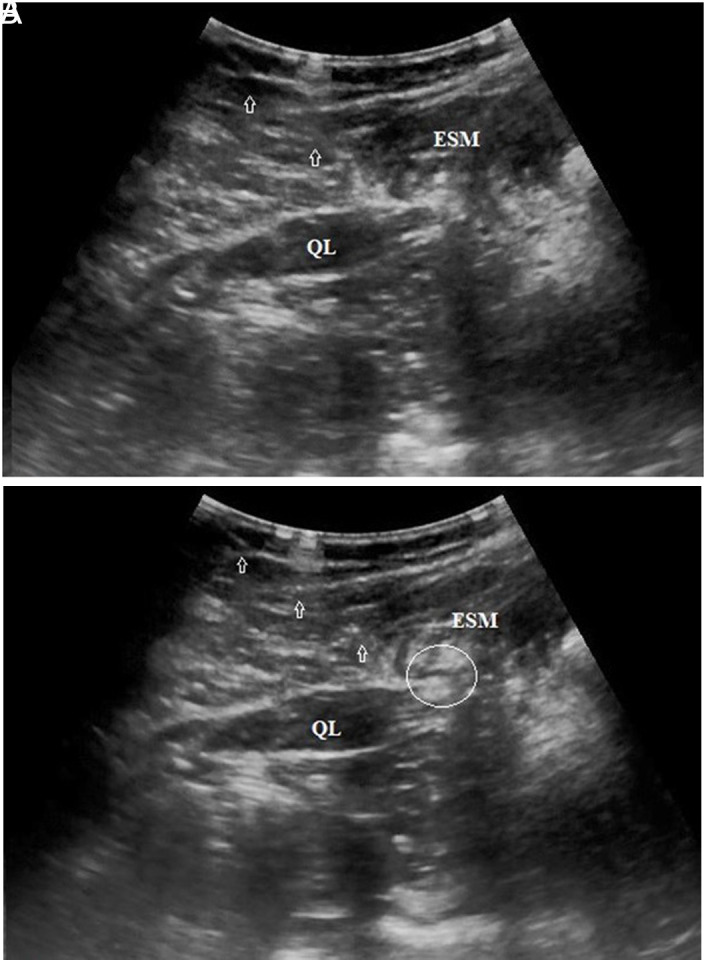
Ultrasound imaging interfascial injection. 2A, 2B: QL: quadratus lumborum muscle, ESM: erector spinae muscle, white arrows show block needle. 2B: white circle injection site.

Interfascial injection: Before the IFI application, skin preparation was made with the patient in the prone position. An ultrasound (convex probe, 2-6 MHz) was positioned on the midsagittal line in the transverse position, revealing the vertebral processes of the L5 and L4 vertebrae in sequential order. The probe was then directed laterally to the level of the L4 vertebra, allowing for differentiation of the transverse process of the L4 vertebra, as well as the quadratus lumborum (QLB), erector spinae (ES), and latissimus dorsi muscles, and the middle and posterior layers of the thoracolumbar fascia. A 22-G nerve block needle was introduced lateral to the probe using the in-plane technique and proceeded to the middle layer of the TLF between the ES and QLB muscles. After confirming the insertion with hydrodissection (observing that 2 fascia sheets are separated with the injection of 2 mL LA), 10 mL Local anesthetic (LA) (bupivacaine 0.25%) and methylprednisolone acetate (40 mg) were injected (Figure 2). Injection success was confirmed by loss of sensation using the cold test on the lateral and anterior abdominal walls 30 minutes after the procedure. All injections and blocks were performed by the same researcher (KÖ).

### Outcome Measures

Primary outcomes: NRS scores pretreatment (PRT) and at first, second, fourth, and sixth months posttreatment (PST).

Secondary outcomes: ODI scores PRT, and at first, second, fourth, and sixth months PST, monthly consumption of NSAIDs was queried (units/month).

### Statistical Analysis

Descriptive data were presented as frequency, mean, percentage, and standard deviation. Qualitative data were compared using the *χ^2^* test. The data’s normal distribution underwent assessment through the Shapiro–Wilk test, demonstrating normal findings. Between-group comparisons were conducted utilizing an independent samples *t*-test, while paired sample *t*-tests were utilized for intragroup comparisons. Values below α = 0.05 were considered significant indicators of differences between the groups. IBM SPSS 21.0 (IBM SPSS Corp.; Armonk, NY, USA) Statistical Package for the Social Sciences Statistics software was used for statistical analysis.

According to the results of the 10-patient pilot study, the NRS score at 6 months after injection was 3.7 ± 1.8 in patients who received injections. In this study, a power analysis was performed to estimate the prognosis of a 1-point decrease in the NRS score at 6 months postinjection. To achieve 85% power (α = 0.05), 62 participants were needed, with 31 participants per group.

## Results

**Figure 3.**
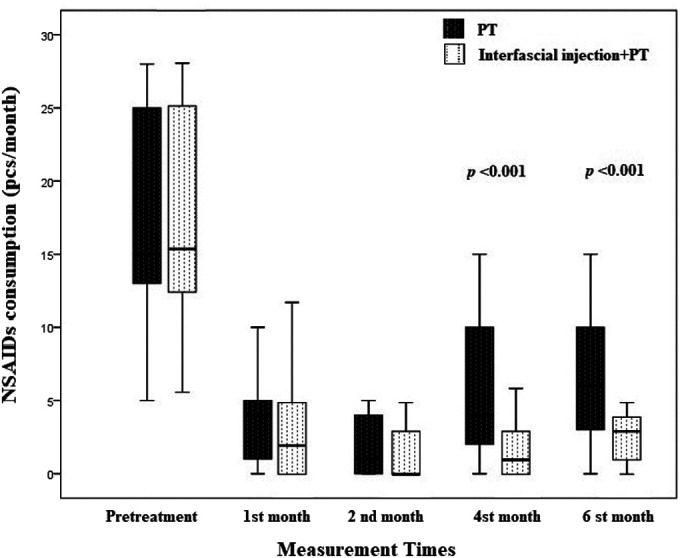
Analgesic consumption.

One participant in the PT group withdrew from the study during the treatment and follow-up phases, out of a total of 62 participants. Therefore, statistical analyses were made with 30 patients in Group PT and 31 patients in Group PT + interfascial injection. The data of 61 patients who were eligible for this clinical study were statistically analyzed. Patients evaluated in the pilot study were not included in this study. There was no statistically significant disparity detected when examining the demographic information of the 2 groups (Table 1). The baseline NRS and ODI scores did not differ significantly between the 2 groups. When the NRS scores in both groups are evaluated, according to the initial values first, second, fourth, and sixth months NRS scores were found to be statistically significantly lower. (Table 2) In the comparison between the groups, the patients in the PT and IFI groups had first, second, fourth, and sixth months NRS scores that were statistically significantly lower than the patients in the PT group (Table 2). The ODI scores were significantly lower than baseline values at first, second, fourth, and sixth month follow-up values in both groups. The ODI scores of the patients in the PT and IFI groups showed a statistically significant decrease in the second, fourth, and sixth months when compared to the patients in the group who received only PT (Table 2). When comparing monthly NSAID usage amounts, the PT + IFI group showed statistically significant lower usage levels at all follow-up times (Figure 3).

## Discussion

The results of this study showed improvement in pain and function at the first-, second-, fourth-, and sixth-month follow-ups of patients who received IFI injections in addition to PT. Improvements in NRS scores from the first month and in ODI scores from the second month were detected.

Previous research has explored both invasive and non-invasive treatment strategies for managing chronic lower back pain. Spinal structures such as the facet joints, intervertebral discs, and annulus fibrosus often contribute to lower back pain, and the fascia and muscles are often overlooked as potential sources of pain.^9,10^ There is a lack of sufficient data on the contribution of TLF to LBP and its place in treatments. There is mounting evidence indicating the involvement of both muscles and fascia in the initiation of lower back pain.^6-8^ This study has addressed the role of TLF in chronic LBP, which has an important place in the anatomical structure of the lumbar region. The TLF is a membrane that comprises aponeurotic and fascial layers, creating a partition between the posterior paraspinal and abdominal wall muscles. The TLF also acts as a component of the myofascial girdle that envelops the lower trunk, contributing to posture, load transfer, and lumbar spine stabilization. The TLF forms a part of the myofascial belt that aids in maintaining posture, transferring loads, and stabilizing the lumbar spine while enclosing the lower body. The middle layer of the TLF that received the injections in the present study is an intermuscular septum that separates the developmental hypaxial and epaxial muscles. The paraspinal retinacular sheath formed by this septum is a hydraulic amplifier that supports the lumbosacral vertebrae and assists in the movement of the paraspinal muscles.^5^ Previous anatomical and histological studies in the literature have focused on the role of TLF in the psychopathology of chronic LBP.^5,10,11^

An immunohistochemical study conducted by Yahia et al^7^ in 1992 identified the presence of free nerve endings and 2 types of encapsulated mechanoreceptors, namely the Vater-Pacini and Ruffini corpuscles, in the TLF. The current evidence suggests that the TLF could potentially serve as a neurosensory component within the lumbar spine mechanism. These results indicate that TLF indeed plays a neurosensory role in the lumbar spine.^5^ In addition, a layer of hyaluronic acid has been described within the loose connective tissue between the fascial layers in muscle fascia, produced by specific types of fibroblasts called fasciacytes.^10^ In an animal study, it was found that stimuli from the TLF became prominent, particularly in the dorsal horn neurons, in pathological conditions that occur as a result of the stimulation of the receptors in the fascial tissues. The findings of the authors have led them to conclude that the TLF plays a crucial role in the provision of nociceptive input to patients suffering from chronic low back pain.^11^ Different results were identified in 2 studies analyzing the effect of TLF on LBP. Kuslich et al^12^ found that the mechanical stimulation of the compressed nerve root causes symptoms of back pain, whereas a similar stimulation of the posterior layer of the TLF produces no such symptoms. The authors reported that stimulation of the posterior layers of the TLF without nerve root compression induced pain in only 32 out of 193 patients.^12^ In a similar study, isotonic saline (0.9%) or hypertonic saline (5.8%) was injected into the erector spinae muscle, the posterior TLF, and the subcutaneous tissue in 12 healthy patients, and pain intensity, duration, quality, and radiation were evaluated. It was found that the pain occurring after the fascial injection was more intense than the pain induced by injection into the muscle. When pain descriptors (burning, throbbing, and penetrating) after fascial injection were evaluated, it was found that pain was conducted by both nociceptive A and C fibers. Based on these findings, it was reported by the authors that TLF might play a role in non-specific LBP and that this area was particularly susceptible to chemical stimulation.^4^ On the other hand, TLF has been shown to be related to the disc disease in the adjacent lumbar segment due to the bulging occurring in the parasagittal plane, identified through magnetic resonance imaging. That said, it is unclear whether this observation shows a predisposition to a pathology or vice versa.^13^

There is limited literature on the effect of fascia injections on low back pain. In case-control studies, erector spinae and thoracolumbar interfascial plane blocks were tested to treat low back pain. The results of both block applications were successful in treating low back pain.^14,15^

In our study, IFI aimed to reduce the pain caused by facial tissue disorders. Blanco et al^16^ reported that the blockade of sympathetic and sensory fibers in TLF was influential in the mechanism of action of fascia blocks used to provide postoperative analgesia. Mense et al^17^ found that TLF may have a nociceptive function due to its free nerve endings and postganglionic sympathetic fibers, which also have a vasoconstrictive function.

We think there are several reasons for the role of TLF in low back pain. One of the reasons is that it may be secondary to lumbar disc pathologies, and the other is that microtraumas occurring in muscle tissue may cause inflammation in the fascial structure and cause chronic pain through structural changes. Accordingly, it can be understood that injection of local anesthetics and steroids into the TLF using the IFI technique may suppress inflammation and nociceptive stimulation in the fascial plane.^4,17^ In addition, the proximity of the epidural spread after the Erector spinae plane (ESP) block to the area where we applied IFI, as shown in other studies, suggests that it may effectively reduce pain through a similar mechanism of epidural spread.^18^

Limitations of this study include the lack of a placebo group and long-term results, and the lack of randomization.

In conclusion, the improvement in the NRS and ODI scores of the patients suggests that TLF may have a role in chronic LBP. We believe that the effects of fascial structures on LBP deserve further research.

## Figures and Tables

**Table 1.** Demographic Characteristics of the Patients

	Interfascial injection +PT (n=31)	PT (n=30)	*P*
Gender			
Female	15 (48.3%)	15 (%50)	.428
Male	16 (52.7%)	15 (%50)	
Age (year)	48.2 ± 10	48.9 ± 6.9	.898
BMI (kg/m^2^)	28.3 ± 3.5	29.1 ± 3.4	.301
Disease duration (month)	11.4 ± 2.8	12.3 ± 2.7	.523

Mean ± SD.

BMI, Body mass index; PT, physical therapy

**Table 2.** Comparison of Measure Time Between the Numerical Rating Scale and ODI score of groups

	Interfascial injection + PT (n = 31)	PT (n = 30)	*P*
NRS score	3	3	3
Pretreatment	5.4 ± 1.1	5.3 ± 1	.809
First month	2 ± 1.2	2.8 ± 1.4	**.024**3
Second month	1.3 ± 1.4	3.3 ± 2	**<.001**3
Fourth month	1.7 ± 1.1	3.7 ± 2.2	**<.001**3
Sixth month	2.1 ± 1.2	3.2 ± 2.3	**<.001**3
P*	**<.001**3	**<.001**3	3
Difference	Pretreatment between others	Pretreatment between others	3
ODI score	3	3	3
Pretreatment	37.8 ± 6.7	36.9 ± 6.8	.625
First month	28.9 ± 9.3	31.5 ± 8.5	.256
Second month	11.6 ± 10.7	21.5 ± 12	**<.001**3
Fourth month	13 ± 6.8	19.5 ± 11.6	**<.001**3
Sixth month	15.4 ± 6.9	22.2 ± 11.9	**<.001**3
P*	**<.001**3	**<.001**3	3
Difference	Pretreatment between others	Pretreatment between others	3

Mean ± SD. χ, bold values; *P* < 0.05.

NRS, Numerical Rating Scale; ODI, Oswestry Disability Index; *P*, comparison between groups.

^*^Comparison within groups (paired sample *t*-test).
